# Treatment of Rheumatism with Phylacogen (Report of Cases)

**Published:** 1914-05

**Authors:** W. C. Kennedy

**Affiliations:** Talmo, Ga.


### TREATMENT OF RHEUMATISM WITH PHYLACOGEN

(Report of Cases)

By W. C. Kennedy, M. D., Talmo, Ga.

The phylacogens, as is more or less well known at the present time, are composed of the product of a great variety of bacteria. These bacteria include the pathogenic organisms that are met with in daily practice. This mixture of metabolic substances constitutes Mixed Infection Phylacogen; and the specific Phylacogens such as Rheumatism, Erysipelas, Pneumonia, and Gonorrhoea are made up of equal quantities of Mixed Infection Phylacogen and the products of the bacteria that cause the respective diseases from which the Phylacogen are named.

As is likewise more or less common knowledge, the Phylacogens are based on the theory of mixed infection. It is Dr. Schafer's contention that practically every infectious disease is caused, not only by a specific micro-organism, but also by a variety of other bacteria. Whether or not this theory is true is not of maximum concern to the medical practitioner. What is of most interest to him is simply will the Phylacogens cure?

I have had some experience with these products, especially Rheumatism Phylacogen and I do not hesitate to say that it has been exceedingly valuable. In my first series, I treated approximately forty cases with success in the majority of instances. Before giving a few case reports, as examples of what the physician may expect from Phylacogen therapy, I wish to take up a few points upon which the successful administration of the Phylacogens depends.

As a factor of great importance I would put the question of diagnosis, and especially does this apply to the treatment of rheumatism. Before the advent of specific bacterial products for the treatment of disease, all that was necessary to treat a case properly was to make a diagnosis of rheumatism. Unfortunately this term covers such a multitude of conditions that considerable confusion arises when such a diagnosis is made. The word rheumatism, as commonly employed, includes not only acute, subacute, and chronic arthritis, but is frequently called upon to cover all sorts of pains in almost every part of the body.

The use of Rheumatism Phylacogen, on the other hand, requires that a specific diagnosis be made. It is generally accepted, although there are a few dissenting voices, that true rheumatism is caused by a specific streptococcus; and it is against this micro-organism that Rheumatism Phylacogen should be directed. If we may judge from cases treated with this preparation, successful and frequently wonderful, results are obtained when the Phylacogen is called upon to combat a true rheumatic infection. An interesting fact in this connection is that it has been reported that Dr. Schafer, the originator of Phylacogen, has made the assertion that if Rheumatism Phylacogen, properly used, fails to benefit a given case, it is bona fide evidence that the case is not one of actual infection by the micro-organism of Poynton and Paine. However that may be, the fact still remains that it is necessary to make an accurate diagnosis.

Probably the most common diagnostic error that is made is the confusion of rheumatic arthritis and gonorrheal arthritis. The physician should not be too willing to accept as final the denial of the patient that he has been afflicted with venereal disease. For obvious reasons, patients are not always willing to confess to this form of infection. Therefore the medical attendant, while accepting at face value the statement of the patient, should hold his mind open, and, in the event that he does not secure beneficial results from Rheumatism Phylacogen, he should reconsider his diagnosis, and possibly even resort to Gonorrhoea Phylacogen as a therapeutic test.

It will be sufficient to mention briefly certain other diseases that must be definitely excluded, without attempting to give the diagnostic points by which each may be distinguished. Tubercular arthritis, gout, flat foot, neurotic arthritis, syphilitic infection, and even unrecognized fracture have each been the cause of failure to secure results from the administration of Rheumatism Phylacogen. Especially tuberculosis and syphilis, in their protean manifestations, have been prolific sources of error. If the usual clinical signs and symptoms are not sufficient to distinguish these diseases, they may be recognized by the application of the tuberculin and Wasserman tests respectively. With the establishment of diagnosis the physician may turn his attention to the details of treatment.

I shall make no attempt to explain the action of Rheumatism Phylacogen; but shall mention briefly a few of the phenomena attending its administration.

After an injection there frequently follows a reaction on the part of the body, manifested by certain distinct symptoms. This reaction is a sequal of large doses subcutaneously, or of almost any sized dose if given intravenously. The symptoms consist of chill, nausea, headache, depression, a rise in temperature, diarrhea, and profuse perspiration. This symptom-complex must not be understood as being complete in each case. Almost any combination of symptoms occur, but the chill, headache, rise in temperature and pulse rate, and profuse perspiration are practically constant. The nausea, depression and diarrhea vary considerably in different patients. But little attention need be paid to this reaction, as it occasions no harm and is frequently in proportion to the degree of benefit that is to be derived from the injection. It is useful also because it indicates the quantity of Phylacogen to be injected in subsequent doses. The physician should aim to produce a fairly marked reaction without allowing the symptoms to become excessive.

As a general rule, injections should be made daily. In the event, however, of an especially intense reaction, an interval of thirty-six to forty-eight hours may be permitted to elapse before the next dose is administered; although only in exceptional instances is it advisable to wait more than forty-eight hours for the reaction to subside. This observation is necessary to avoid a possible exacerbation of the infectious process.

The technique of intravenous injections is by no means formidable. All required is that the strictest possible asepsis be observed, and the careful preparation of the syringe so that no air may be forced into the general circulation. After having a number of intravenous injections the procedure finally becomes no more difficult than the giving of an ordinary subcutaneous injection.

The detailed reports which I shall present have been selected as examples of what may be expected of Phylacogen, the technique and the general management of treatment; and differs to no great extent from other cases which I have treated.

Case 1, aged 52. History: Rheumatic attacks for twenty-five years; one attack of sciatica lasting six months, during which time patient was confined to bed and house. The present attack had been affecting patient all through the winter months.

Diagnosis: Muscular rheumatism.

Rheummatism Phylacogen was begun April 6th, 1912, ½ Cc. being given intravenously. In twenty minutes patient had a chill lasting fifteen minutes, followed by nausea, headache, depression, weakness, a rise in temperature of two degrees, and profuse perspiration.

| | | | | |
|---|---|---|---|---|
| April | 7th, | ½ Cc. | Rheumatism | Phylacogen administered |
| " | 8th, | ¾ Cc. | " | " |
| " | 9th, | 1 Cc. | " | " |
| " | 10th, | 1½ Cc. | " | " |
| " | 11th, | 2 Cc. | " | " |

This dose was followed by a severe chill lasting thirty minutes, and such a general reaction that on April 12th, 1Cc. Phylacogen was given followed by a slight general reaction. April 13th, 1 Cc. Rheumatism Phylacogen. April 14th, 1 Cc.; 15th, 1 Cc.; 16th, 1 Cc.; 17th, 1½ Cc. All the doses were followed by chill and general reaction except the last three doses. In one week patient resumd work in the shop. It is now eleven months since treatment was discontinued and patient still remains immune from rheumatism and states that his health has been better during the past year than it has been in twelve years despite his age.

Case 2. Patient, aged 36. Family history good. History of rheumatism for past ten years. For past five years patient has suffered continually from pain in joints of back, arms, shoulders, and legs, with some enlargement of joints of hands and feet.

Diagnosis: Rheumatic arthritis.

June 1st, 1912, Rheumatism Phylacogen, ½ Cc. intravenously was administered. June 2nd, 1 Cc. Rheumatism Phylacogen; June 3rd, 1 Cc.; June 4th, 1 Cc.; June 5th, 1½ Cc.; June 6th, 1½ Cc.; June 7th, 1½ Cc.; June 8th 2 Cc.; June 9th, 2 Cc.; June 10th, 3 Cc.; June 11th, 3 Cc.; June 12th, 3½ Cc.; June 13th, 4 Cc. All doses, except the last three, were followed by chill and general reaction. Rheumatism Phylacogen discontinued. Patient is now free from pain; general health beginning to improve; eating heartily and sleeping well. This patient had previously suffered from dyspepsia, and can now eat any form of diet. Discharged feeling well. Patient received in all 25½ Cc. of Phylacogen.

September 1st, 1912, patient returned with the rheumatic condition in joints of hands and feet, and wanted another course of the same treatment. September 1st, 5 Cc. Rheumatism Phylacogen were given subcutaneously. September 2nd, 10 Cc., subcutaneously. Each day until September 18th 10 Cc. were administered with the exception of the 15th and 17th. A total of sixteen doses, amounting to 155 Cc. were given. The doses on the 16th and 18th were not followed by any local or general reaction; all the other doses were followed by chill, nausea, rise in temperature, and general reaction. Patient is now free from pain and was discharged feeling well.

January 1st, 1913, patient's general health better than for years, but still has some tenderness in wrist and finger joints.

Case 3. Patient, Mrs. L., aged 43, gave history of having suffered from rheumatism for past ten years, in first one joint and then another at intervals of three or four months. June 15th, 1912, I called to see patient, who had badly swollen ankle which was very tender to the touch. Temperature 102, pulse 100. Had been in this condition four days.

Diagnosis: Acute articular rheumatism.

June 15th, I gave 2 Cc. Rheumatism Phylacogen subcutaneously, which was followed by slight chill, local and general reaction. June 16th, 2 Cc. Rheumatism Phylacogen were administered. June 17th, 3 Cc.; June 18th, 4 Cc., with slight reaction and no chill. Patient much encouraged, and able to bear weight on foot without pain; all swelling gone from ankle. Temperature and pulse normal. June 19th, 5 Cc. Rheumatism Phylacogen. June 20th, 5 Cc. Neither of the last two doses were followed by any local or general reaction. Patient discharged well July 1st, and has had no return of the rheumatic condition.

Case 4. Male, aged 27. History of rheumatic attacks for past five years. The present attack extended over a period of two months. Patient was unable to move or help himself in any way, and the slightest jar would send him into paroxysms. Every joint in his body was more or less affected. Temperature 102, pulse 120, and very weak; his general health was very bad; weight before present attack was 150 lbs., now 110 lbs. The patient had taken anti-rheumatic treatment from beginning of attack with little or no benefit.

Diagnosis: Subacute articular rheumatism.

July 1st, 1912, I gave ½ Cc. Rheumatism Phylacogen intravenously, which was followed in thirteen minutes by chill lasting thirty minutes, with nausea, headache, rise in temperature, profuse perspiration, depression and weakness. July 2nd, ½ Cc. Phylacogen was given, with reaction lighter; July 3rd, 1 Cc. July 4th, 2 Cc.; July 5th, 2 Cc.; July 6th, 2 Cc. All the doses were followed by chill and general reaction. Patient was able to turn himself in bed, and take some nourishment. Decided to let patient have a two-weeks' rest to gain strength. July 21st patient was able to come to office in wagon, a distance of four miles. I then gave 5 Cc. Rheumatism Phylacogen subcutaneously, with local and general reaction following. July 22 nd, 10 Cc. Phylacogen; July 23rd, 10 Cc. Phylacogen; July 24th, 10 Cc.; July 25th, 10 Cc. All of the doses were followed by local and general reaction. July 26th, 12 Cc. were administered. No chill or general reaction followed. Two weeks later patient had recovered from stiffened condition of muscles that follows the administration of Rheumatism Phylacogen. September 20th, patient able to return to work, was gaining in strength and free from the rheumatic condition. Until February 1st, patient still remained immune.

Case 5. Farmer, aged 30. History of rheumatism for past ten or twelve years. Present attack of one week's duration; patient unable to do any kind of work or dress himself on account of the pain in muscles, arms, shoulders, back and legs.

Diagnosis: Muscular rheumatism. Patient decided to take Phylacogen treatment. January 13th, 1913, I administered 10 Cc. Rheumatism Phylacogen subcutaneously, with 10 Cc. each day until six doses had been given, each dose being followed by local and general reaction. Three days after last dose patient resumed work and has not lost a day since. Has been out in all kinds of weather this winter, since taking the Phylacogen, and feels better than he has for years. So far remains immune against rheumatic infection.

Results in the foregoing cases were certainly not coincidences. In some instances the usual treatment had been tried without success and the patients were in pitiable condition, suffering extremely, and totally incapacitated. They were almost invariably well satisfied with he results of treatment and in practically no case was there serious complaint in regard to the reaction following the injection. It is possible that, if a more protracted course of treatment had been followed out, in case two the patient would not have had the slight return of the disease which occurred about three months later. The second attack, however, yielded nicely to the Phylacogen and the patient was discharged practically free from his difficulty with the exception with the exception of slight tenderness in wrists and finger joints.

There is only one conclusion that can be drawn from these cases, which is, that Rheumatism Phylacogen is of exceedingly great therapeutic value. It is not to be expected that every case will yield to its influence; nevertheless, when a failure results, the physician should not be content in the belief that this happens to be a case in which Rheumatism Phylacogen will not cure, but he should reconsider his diagnosis and see if a tubercular infection or a gonorrheal infection, or possibly an obscure syphilitic infection is not really the cause instead of the streptococcus rheumaticus.

To sum up, it may be stated that physicians with the greatest experience are most favorably inclined towards Phylacogen. I believe that every patient should be given the benefit of this treatment, and especially is this true if other remedies have been tried in vain. If results continue to be as satisfactory as they have been, Rheumatism Phylacogen has before it a satisfactory if not wonderful future.

